# Greatest of All Time: Development of a Ranking System to Determine the Most Successful Olympic and World Championship Runners Since 1896

**DOI:** 10.1007/s40279-026-02443-2

**Published:** 2026-05-09

**Authors:** Brian Hanley, Carl Foster, Arturo Casado, David B. Pyne, Andrew M. Jones, Renato Barroso, Daniel Boullosa, Jos J. de Koning, Thomas Haugen, Florentina J. Hettinga, Andrew Renfree, Stephen Seiler, Alan St Clair Gibson, Philip Skiba, Christian Thiel, Randall Wilber

**Affiliations:** 1 Leeds Beckett University, Leeds, UK; 2 University of Wisconsin-La Crosse, La Crosse, USA; 3 Rey Juan Carlos University, Madrid, Spain; 4 University of Canberra, Canberra, Australia; 5 University of Exeter, Exeter, UK; 6 Universidade Estadual de Campinas, Campinas, Brazil; 7 University of León, León, Spain; 8 Vrije Universiteit-Amsterdam, Amsterdam, The Netherlands; 9 Kristiania University of Applied Sciences, Oslo, Norway; 10 University of Worcester, Worcester, UK; 11 University of Adger, Kristiansand, Norway; 12 University of Aberdeen, Aberdeen, UK; 13 Thomas Jefferson University – Sidney Kimmel College of Medicine, Philadelphia, USA; 14 Bochum University of Applied Sciences, Bochum, Germany; 15 USOC, Colorado Springs, USA

## Abstract

**Background:**

This study aimed to identify the Greatest of All Time (GOAT) athlete in running events held at the Olympic Games and World Athletics championships.

**Methods:**

The achievements of 1294 men and 824 women who won at least one medal in any sprint, hurdles, or distance event at major global championships since 1896, or set a World Record (WR) since 1912, were collated. A scoring system was used to award points, with Olympic gold medals and WRs ranked joint highest. Fewer points were awarded for Olympic silver and bronze medals and for medals in World Championships (outdoor, indoor, and cross-country). Bonus points were awarded for WR longevity and the setting of WRs during Olympic finals. Athletes were also ranked by event and within historical eras.

**Results:**

As of March 2026, Usain Bolt (Jamaica) was ranked as the male GOAT, and the highest-scoring woman was Faith Kipyegon (Kenya). Both athletes notably won “doubles” during their careers (over 100/200 m and 1500 m/5000 m, respectively) and set WRs over both distances. Marathon, steeplechase, and hurdle specialists usually competed in one event only. In an earlier era, Paavo Nurmi (Finland) had the highest combined Olympic Games/WR score across a range of distances.

**Conclusions:**

Athletes who successfully competed over physiologically and tactically similar events (“doubles”), such as Bolt and Kipyegon, had a greater opportunity to become the GOAT. An increase in global competition since 1972 meant greater opportunities for winning medals, especially for women. This study provides a foundation on which future performances could be evaluated.

**Supplementary Information:**

The online version contains supplementary material available at 10.1007/s40279-026-02443-2.

## Key Points

**Table:** 

A ranking system was developed to evaluate the performances of all runners who have won a medal at the Olympic Games, World Athletics Championships, World Athletics Indoor Championships, or World Cross Country Championships or have set a WR at the standard track distances (and marathon).
Points were allocated on the basis of the current prize money awarded by World Athletics, which we feel is organic and tied to the intrinsic value of different competitions. Athletes who also competed in relays might have been undervalued, as only individual events were included.
Jamaican sprinter Usain Bolt was the male GOAT, having won six individual Olympic gold medals and seven individual World Championship gold medals and holds the 100-m and 200-m WRs. Faith Kipyegon was considered the female GOAT, having won three Olympic gold medals, five World Championship gold medals, and is the current holder of the 1500-m WR, as well as having previously held the 5000-m WR.
Because of differences in the availability of competition over the 124 years of the modern Olympic Games, athletes’ performances were assessed within identified historical competitive “eras.” Paavo Nurmi, winner of Olympic medals at the 1920, 1924, and 1928 Games in the 1500 m, 3000 m steeplechase, 5000 m, 10,000 m, and cross country and holder of WRs at 1500 m, 5000 m, and 10,000 m, was the standout performer of the early Twentieth century.

## Introduction

In 1894, Pierre de Coubertin organized the modern Olympic movement around the concept of *citius, altius, fortius* (“faster, higher, stronger”) [1], borrowed from Henri Didon, a Dominican priest who taught sport near Paris [1]. Since the first modern Olympic Games, this concept has defined our quest for perfection in sport, with more contemporary values expressed in terms such as excellence, respect, and friendship. Despite many flaws, scandals, doping, commercialism, and elitism [2], as well as cancellations because of two World Wars and considerable political interference [3], the Olympic movement remains one of the most remarkable ideas humankind has ever had for promoting sport, culture, and education. In recent decades, the structure and concept of the Olympics have been extended to World and continental championships and to cross-country and indoor competitions.

At its simplest, this quest for excellence centers around medals won in Olympic Games (OG) or World Championship (WC) competition or World Records (WRs) achieved. Although the relative merits of global medals and WRs are debatable, they are frequently the main parameters of success when comparing athletes across generations and specialties when intending to award the unofficial title of Greatest of All Time (GOAT) within any given sport [4–6]. Many sports do not readily lend themselves to defining the best individual, as they depend on team play [7], and are not inherently quantitative. By contrast, athletics is highly quantitative, with distance and duration as precisely measured variables, lending itself to identification of a GOAT. Of course, the Olympic program changes over time and the potential size of the athletic population is steadily increasing, attributable to increased human participation in sport and more countries participating in the Olympic movement. Nonetheless, championship medals and WRs provide strong parameters for measuring sporting success [8] and in setting outcomes for defining the GOAT.

Beyond the intrinsic enjoyment of fans debating how to determine the identity of the GOAT, the process can also lead to conceptually analyzing sport, with the potential to inform data-driven evaluation of sports performance more broadly. This study represents the fruits of a quantitative discussion by 16 of us, predominantly scientists rather than elite athletes or coaches, to identify the GOAT in running. To aid this process, Florentina Hettinga, as part of a presentation at the 2024 European Endurance Conference, informally surveyed 63 coaches, athletes, and sport scientists using a show of hands on how to determine the GOAT in running. The conference delegates were shown the questions on a screen, and each question was debated in the context of the conference. When asked which of two criteria they believed was of more value relative to determining the GOAT, 71% favored becoming Olympic Champion whereas 29% favored setting a WR. Regarding minimum thresholds, there was a more even split regarding whether the GOAT had to have been an Olympic champion (yes: 54%, no: 46%). Respondents were very much (95%) in favor of identifying a GOAT per main running subdiscipline (e.g., sprints, hurdles, or distance) and in identifying a GOAT in men’s and women’s running separately (95%). Respondents were unanimously in favor of eliminating any candidate who had been banned for doping.

Thus, taking into account these differences of opinion regarding the relative importance and value of OG and WC medals and WRs, the research team considered there were several potential contributing factors for identifying the GOAT runner:

- Outstanding performance reflected by WR performance(s), particularly WRs that went unbroken for many years or set multiple times by the same athlete. It was not considered necessary for an athlete to have set a WR to be identified as the GOAT (overall or in any subcategory).
- Outstanding performance reflected by medals won in major global championships (OG or WC). We decided to match the points awarded for Olympic gold medals and WRs as an acknowledgement that, whereas Olympic gold medals theoretically reflect racing excellence, WRs were an equivalent reflection of physiological and performance superiority.
- Outstanding performance in smaller global championships, specifically the World Cross Country Championships and the World Athletics Indoor Championships.
- Competitive dominance reflected by the range of competitive distances, particularly “doubles” or “triples” where the athlete medaled in more than one event at a single OG or WC and as reflected by the length of their career, which was quantified by the number of medals won or WRs achieved. We did, however, not factor in how many years each athlete actively competed for or how many championships they competed in.
- We decided to include all performances that are currently accepted as valid by World Athletics, regardless of previous or later doping offences, given that these performances are officially sanctioned.

Each of these factors is quasi-independent, reflecting differences in physiology [9, 10], competitive goals, avoidance of competitive disruptions secondary to lack of opportunity, injury, war, boycotts, and other factors that affect the ability to compete [11]. Accordingly, the purpose of this exploratory study was to develop an objective assessment of the running GOAT by analyzing historical performance data of running events contested at the OG or WC (including indoor and cross-country events). We sought insights regarding extremely talented and highly trained athletes in terms of optimal physiology as they perform at the limits of human performance, as well as examining whether specific events meant a greater likelihood of being identified as the GOAT. We evaluated the winning of medals at major championships and the setting of WRs in the context of the opportunities available to elite runners during their competitive lifetimes using a custom-made normative scoring system that was intended to be organic and framed in the language of athletics, rather than physiology.

## Methods

### Participants

The study was approved by an institutional review board (application no. 144192) on the basis that all data were openly available. An observational design, using data from online sources (https://worldathletics.org), was used to rank and evaluate the achievements of athletes competing in running events held as part of the Olympic program. Athletes were included if they had won at least one medal at a major global championship [12] or set a WR ratified by World Athletics [13] as of 1 March 2026. World Best marathon times [13] from before 2004 were considered WRs provided they were accepted by the International Association of Athletics Federations (IAAF), since renamed World Athletics. As the IAAF was founded in 1912 with a purpose of ratifying WRs [14], we excluded WRs set before 1912. Analyzed athletes comprised 1294 men and 824 women who competed in sprint, distance, and hurdling events. These included obsolete events such as the 60 m, 200-m hurdles, various steeplechase races, women’s 80-m hurdles (replaced by 100-m hurdles) and women’s 3000 m (replaced by 5000 m). Performances in team events like relays and the obsolete 3000-m team race were excluded. Those athletes who won medals in the three editions of the Olympic men’s individual cross-country (1912–1924) were incorporated in the tally for the World Cross Country Championships (WXC). Athletes’ nationalities were indicated using their World Athletics member federation codes [13], thereby showing the names of federations that existed at the time. Data were collated and points calculated using Microsoft Excel; conditional formatting was used to check for duplicate entries, and online sources (https://en.wikipedia.org/wiki/, https://www.olympedia.org/) were used to double-check athlete performances. This was particularly useful for identifying those athletes who won medals under different surnames or for more than one nation.

### Data Analysis

Points were awarded for medals on the basis of the value of prize money awarded by World Athletics for the 2025 World Athletics Championships, 2025 World Indoor Championships, 2026 World Cross Country Championships, and World Records (set during the 2025 World Championships) [15–17], regardless of what year the competition was held in. We awarded 1 point per US $1000 of prize money as presented in Table 1. No prize money is awarded in the same way for the Olympic Games, so we decided to use 100 points for a gold medal so that it matched the WR points system and used the same ratios for silver and bronze medals used for the World Championships (Table 1). Although World Athletics did pay US $50,000 to gold medalists in Paris 2024 [18], we did not add any extra points to the default 100 points for Olympic victories. A greater points allocation was awarded to Olympic medals than those in the WC given their less frequent occurrence (every 4 years compared with the biennial WC) and their greater importance to national sporting funding bodies [8, 19, 20]. Overall, the approach taken was a descriptive study using the sum of allocated points to determine rankings.

**Table 1:** The normative points system used to calculate athlete scores

Championship	Gold	Silver	Bronze	Other
Olympic Games	100	50	31	
World Athletics Championships	70	35	22	
World Athletics Indoor Championships	40	20	10	
World Cross Country Championships	30	15	10	
World Record				100
World Record + Olympic gold				+ 5
World Record duration				+ 2 per year

WRs were obtained only for those running events that have been part of the Olympic outdoor track and marathon program. WRs for the 200 m were included from 1951 onward, as previous WRs could be set on a straight, rather than a curved track [13]. For women, whose first Olympic athletics competition was in 1928 [21], WRs were taken from when those events were recognized by the IAAF. Women’s WRs were recorded for the 80-m hurdles and 3000 m until their replacement by the 100-m hurdles and 5000 m, respectively. Although the first IAAF WC were inaugurated in 1983, the women’s 400-m hurdles and 3000 m from the 1980 World Championships in Athletics were included. Similarly, we incorporated the IAAF World Indoor Games (1985) with the World Athletics Indoor Championships (WIC). We also included the indoor 200-m race in our analysis, which was discontinued after 2004. We included women’s marathon WRs from 1970 onward, as this was the period when women’s marathons became formalized [22], and points were awarded for WRs set in women-only races. Given the truly outstanding nature of setting a WR during an Olympic final, 5 bonus points were awarded to any athlete achieving this feat. To factor in the quality of a WR, a longevity bonus was awarded: for each full year that a WR was held by the same athlete, an additional 2 points were awarded. In those cases where a particular athlete broke their own WR, or held it on multiple occasions (i.e., regained it from another athlete), the cumulative duration of their WR durations was used. Joint WRs were common in the sprint events when hand-timing was used; these were all considered WRs. All points awarded for medals and WRs were based on the official results for those events; athletes whose medals or WRs were subsequently rescinded were not awarded points for those achievements. Athletes who were banned for a period of time for doping violations were included in the sample for any results that were ratified despite the ban.

To aid in establishing the robustness of the model used, a test of sensitivity analysis was conducted. In this process, several different scenarios were run where the contribution of particular achievements were adjusted to identify what changes would occur in the ranking of the top 10 men and women. These scenarios were: (1) an increase in OG points so that they were double the WC points (because the frequency of OG is half that of WC) and WR points adjusted to 140 points to match the new OG gold score; (2) no bonus for setting a WR during an OG gold medal winning race; (3) no bonus for WR duration; (4) no points awarded for cross country (XC) medals; and (5) no points awarded for WIC medals. Although there are innumerable possibilities for altering the points system, these five scenarios were felt sufficient for testing the overall points structure adopted.

**Fig. 1:**
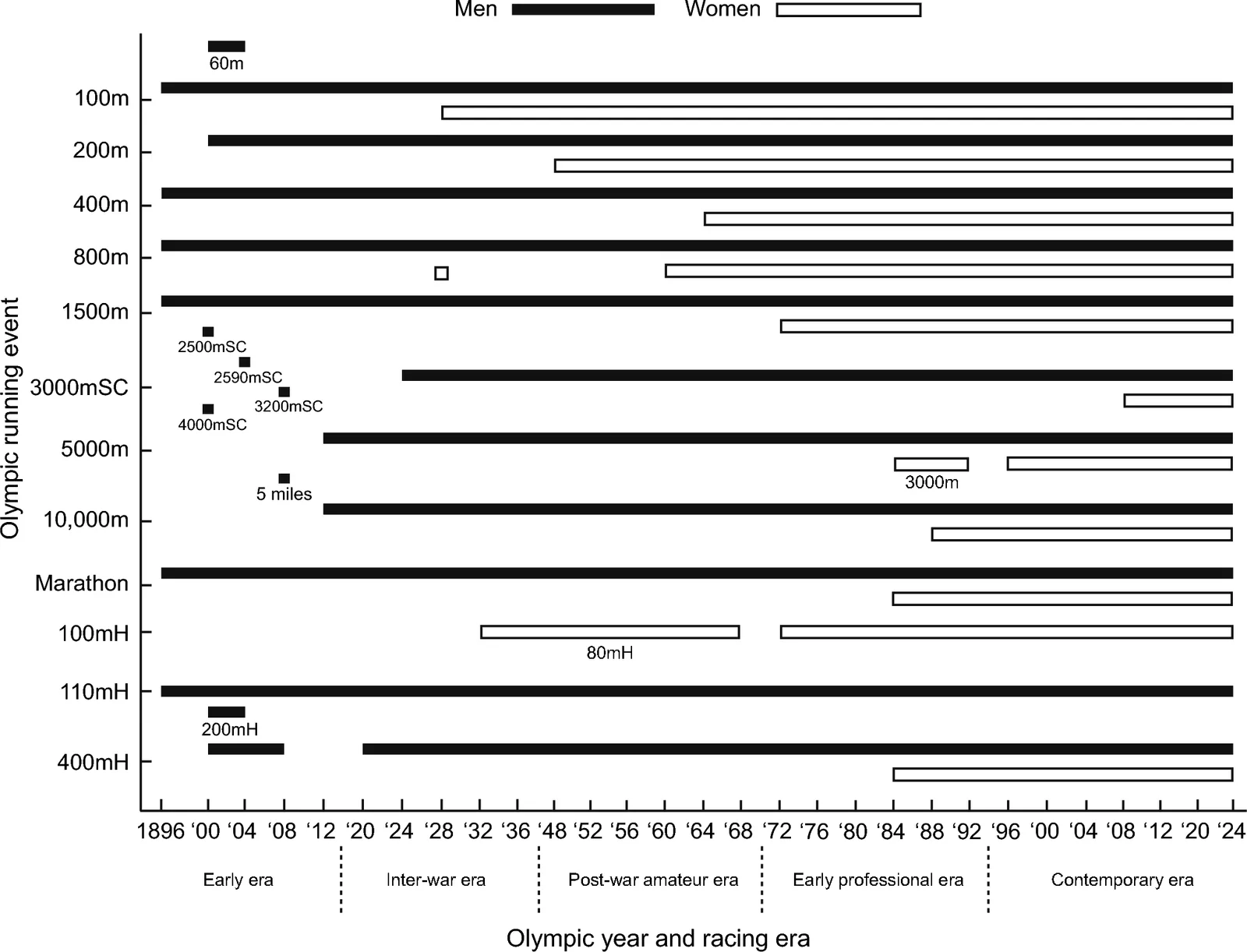
Five identified racing eras for men and women since the inception of the first modern Olympic Games in 1896

To account for historical differences, we defined five distinct racing eras (Fig. 1). These eras overlapped with those identified in a previous historical analysis of Olympic 1500-m performances [23] and are related to the number of athletes competing, the number of championships and events available, the impact of both World Wars, the effect of globalization, and the removal of the amateur code in athletics. Each era was defined by Olympiad years. We grouped the first five OG as the “early era” (1896–1912), which was pre IAAF with only a small number of men representing relatively few nations competing in a nonstandardized range of events. The next five Games (1920–1936) form the “interwar era,” which featured the recognized men’s events that continue today (but included cross-country races) and, from 1928, two running events for women. The Games from 1948 to 1968 were deemed the “post-war amateur era,” where the number of running events for women increased from three to five but when many successful athletes retired early to focus on professional careers [23]. The “early professional” era ran from 1972 to 1992, during which Olympic events for women first included the 1500 m (1972), 400-m hurdles, 3000 m and marathon (1984), 10,000 m (1988), and the 80-m hurdles increased to 100 m (1972). It was during this era that the WC, WXC, and WIC were first held. The final era identified was the current “contemporary era,” starting in 1996, when the women’s 5000 m replaced the 3000 m, the women’s 3000-m steeplechase was introduced (in 2008), and the WXC became a biennial event after 2011. Several recent championships were either postponed or canceled because of the coronavirus disease 2019 (COVID) pandemic [24]. Athletes were allocated to an era on the basis of the first year they won a medal or set a WR, meaning that some spanned more than one era; the total score across eras was used in these instances.

As well as proposing the overall male and female GOATs, and those during each era, we also described the GOAT for event groupings. For the Olympic program, these comprised the sprints (60–400 m), hurdles (80–400 m), middle-distance (800–1500 m), steeplechase (mostly 3000 m but also early variants), long-distance track (5000–10,000 m), and marathon events; we also had categories for cross-country and indoor running (all events). The since-replaced women’s 3000 m was included in the long-distance category. When assessing the GOAT in these categories, only those medals and WRs achieved over the specified distances were included, meaning that events like the 1000 m, mile, and half-marathon were excluded. Because certain events were often “doubled up” by athletes, we compared feasible doubles by pairing race distances by increasing distance, i.e., 100 m and 200 m, 200 m and 400 m, 400 m and 800 m, 800 m and 1500 m, 1500 m and 5000 m, 5000 m and 10,000 m, and 10,000 m and marathon. The number of doubles achieved during the past two eras was calculated and the distances compared for duration, mean running speed, approximate aerobic contribution for men [25–27], and similarity to other championship events.

## Results

**Fig. 2:**
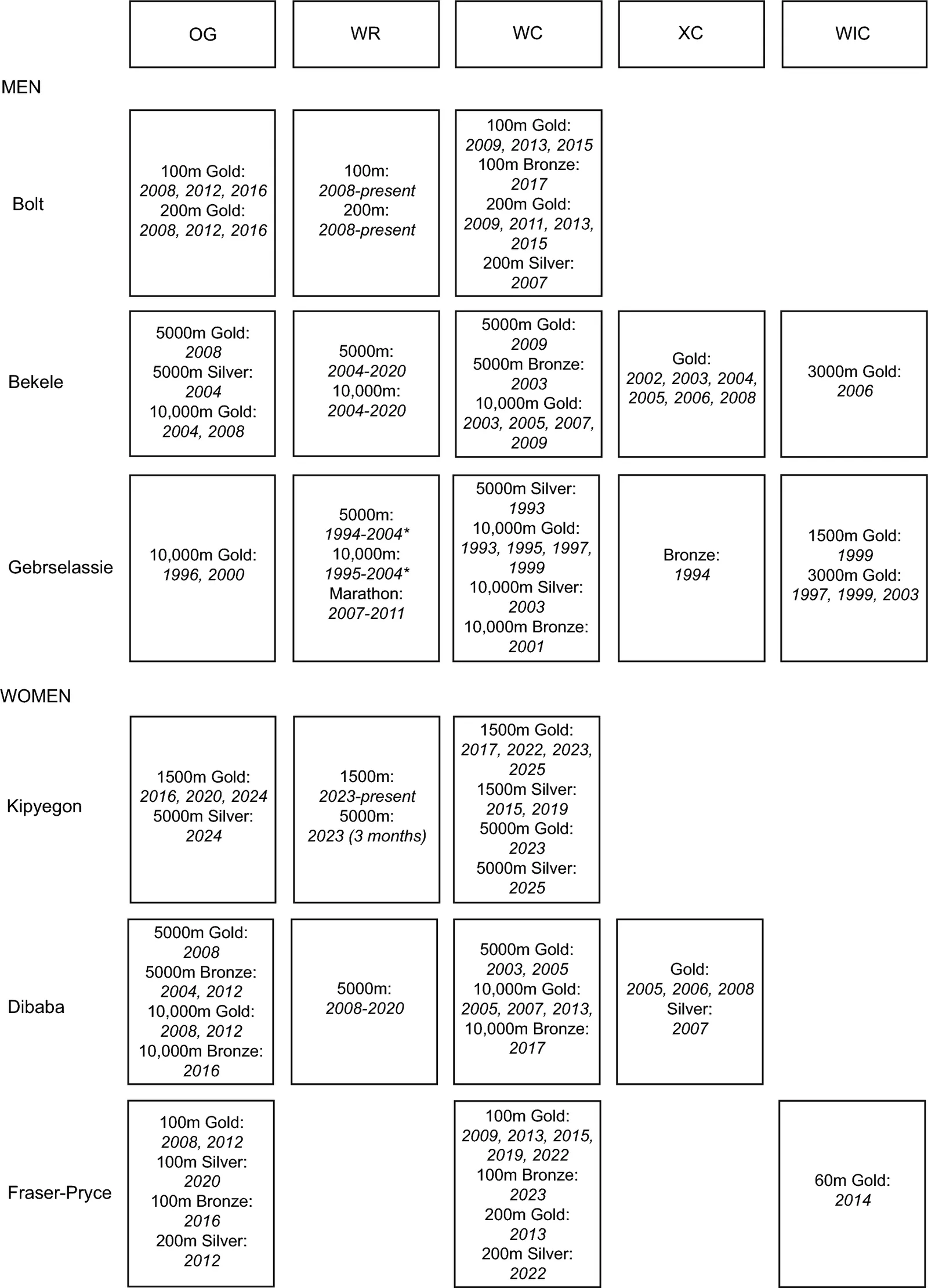
Championship medals won and WRs set by the top three men and women in their respective ranking lists. An asterisk (*) indicates that the athlete lost and reclaimed the WR during this time

The highest-ranked 25 men and women according to our normative scoring system are presented in Tables 2 and 3, respectively. In both groups, 22 of the 25 were Olympic Champion at least once; the exceptions in the men’s list were Paul Tergat, Bernard Lagat, and Moses Kiptanui, and in the women’s list the exceptions were Merlene Ottey, Grete Waitz, and Jarmila Kratochvílová. Amongst the men, Usain Bolt was determined to be the clear GOAT as of March 2026, having won six Olympic gold medals and seven WC gold medals and, since 2009, still holding the 100-m and 200-m WRs. Paavo Nurmi, who was fifth amongst the men, competed in an era before WC, and had more points in OG and WR categories than any of the runners above him. Faith Kipyegon was ranked the highest amongst women, having won three Olympic gold medals, five WC gold medals, is the current 1500-m WR holder, and previously held the 5000-m WR. The medals and WRs accumulated as of March 2026 by the top three men and women in their respective ranking lists are shown in Fig. 2.

**Table 2:** The 25 highest-ranked men used to determine the male GOAT

Rank	Athlete	Event	OG score	WR score	WC score	XC score	WIC score	Total score
1	Usain Bolt (JAM)	SP	600	278	547	0	0	1425
2	Kenenisa Bekele (ETH)	LD	350	264	372	180	40	1206
3	Haile Gebrselassie (ETH)	LD	200	336	372	10	160	1078
4	Michael Johnson (USA)	SP	300	261	420	0	0	981
5	Paavo Nurmi (FIN)	LD	550	352	0	60	0	962
6	Mohamed Farah (GBR)	LD	400	0	490	0	0	890
7	Hicham El Guerrouj (MAR)	MD	250	154	350	0	120	874
8	Joshua Cheptegei (UGA)	LD	250	220	245	40	0	755
9	Carl Lewis (USA)	SP	350	117	232	0	0	699
10	Emil Zátopek (TCH)	LD	450	212	0	0	0	662
11	Justin Gatlin (USA)	SP	212	0	350	0	80	642
12	Lasse Virén (FIN)	LD	400	205	0	0	0	605
13	Ezekiel Kemboi (KEN)	HU	200	0	385	0	0	585
14	Hannes Kolehmainen (FIN)	LD	300	235	0	30	0	565
15	Maurice Greene (USA)	SP	131	110	280	0	40	561
16	Paul Tergat (KEN)	LD	100	208	92	160	0	560
17	Noah Lyles (USA)	SP	162	0	372	0	20	554
18	Allen Johnson (USA)	HU	100	0	302	0	140	542
19	Sebastian Coe (GBR)	MD	300	236	0	0	0	536
20	Ville Ritola (FIN)	LD	400	105	0	15	0	520
21	Jakob Ingebrigtsen (NOR)	MD	200	0	210	0	100	510
22 =	Bernard Lagat (KEN/USA)	LD	81	0	267	0	160	508
22 =	Edwin Moses (USA)	HU	231	137	140	0	0	508
24	Eliud Kipchoge (KEN)	LD	281	110	105	0	10	506
25	Moses Kiptanui (KEN)	HU	50	208	245	0	0	503

Each athlete’s main event grouping is indicated; marathon performances were included in the long-distance events

SP, sprints; HU, hurdles/steeplechase; MD, middle-distance; LD, long-distance; OG, Olympic Games; WR, World Record; WC, World Athletics Championships; XC, Cross country (includes Olympic Games and World Cross Country Championships); WIC, World Athletics Indoor Championships

**Table 3:** The 25 highest-ranked women used to determine the female GOAT

Rank	Athlete	Event	OG score	WR score	WC score	XC score	WIC score	Total score
1	Faith Kipyegon (KEN)	MD	350	204	455	0	0	1009
2	Tirunesh Dibaba (ETH)	LD	393	124	385	105	0	1007
3	Shelly-Ann Fraser-Pryce (JAM)	SP	331	0	477	0	40	848
4	Sifan Hassan (NED)	LD	393	100	241	0	70	804
5	Merlene Ottey (JAM/SLO)	SP	255	0	355	0	180	790
6	Meseret Defar (ETH)	LD	250	104	219	0	210	783
7	Gail Devers (USA)	SP	200	0	350	0	200	750
8	Maria Mutola (MOZ)	MD	131	0	267	0	310	708
9	Tatyana Kazankina (URS)	MD	300	363	22	15	0	700
10	Beatrice Chebet (KEN)	LD	200	202	197	60	0	659
11	Irena Szewińska (POL)	SP	312	336	0	0	0	648
12	Veronica Campbell-Brown (JAM)	SP	262	0	302	0	80	644
13	Allyson Felix (USA)	SP	281	0	359	0	0	640
14	Florence Griffith-Joyner (USA)	SP	250	353	35	0	0	638
15	Vivian Cheruiyot (KEN)	LD	231	0	315	30	20	596
16	Gabriela Szabo (ROU)	MD	181	0	210	0	180	571
17	Marita Koch (GDR)	SP	100	306	105	0	40	551
18	Fanny Blankers-Koen (NED)	SP	300	226	0	0	0	526
19	Shirley Strickland (AUS)	HU	293	215	0	0	0	508
20	Grete Waitz (NOR)	LD	50	208	70	170	0	498
21	Renate Stecher (GDR)	SP	281	213	0	0	0	494
22	Sydney McLaughlin (USA)	HU	200	113	175	0	0	488
23	Tetyana Samolenko (URS)	MD	181	0	245	0	60	486
24	Jarmila Kratochvílová (TCH)	MD	50	288	140	0	0	478
25	Elaine Thompson-Herah (JAM)	SP	400	0	57	0	10	467

Each athlete’s main event grouping is indicated; marathon performances were included in the long-distance events

SP, sprints; HU, hurdles/steeplechase; MD, middle-distance; LD, long-distance; OG, Olympic Games; WR, World Record; WC, World Athletics Championships; XC, cross country (includes World Cross Country Championships only); WIC, World Athletics Indoor Championships

The results for each era are presented in Table 4. Amongst men, the early and interwar eras were dominated by US sprinters and Finnish distance runners, whereas middle-distance (Peter Snell) and long-distance runners (Emil Zátopek, Vladimir Kuts) were the highest ranked during the postwar amateur era. The top-ranked men in the most recent eras were predominantly sprinters (Carl Lewis and Bolt) or long-distance runners (Kenenisa Bekele, Haile Gebrselassie and Lasse Virén). Very few events were held for women in the interwar OG, meaning that few points were achieved, and the top three women from this era scored more from WR performances than from their medal haul. Whereas Irena Szewińska (née Kirszenstein) won Olympic medals across 100 m, 200 m, and 400 m in three separate Games across two eras, Fanny Blankers-Koen won all three available women’s running events in 1948 (100 m, 200 m and 80-m hurdles). In the early professional era, Gail Devers emulated Blankers-Koen in winning 100 m and short hurdles titles but over the course of two OGs and three WC. The second-highest ranked woman during the early professional era, Merlene Ottey, had the largest haul of individual medals: seven Olympic medals, ten WC medals, and seven WIC medals, spanning 1980–2003. The top two ranking spots in the contemporary era went to a predominantly middle-distance and long-distance athlete, respectively, with Kipyegon’s better WC and WR scores putting her ahead of Tirunesh Dibaba on total points.

**Table 4:** The three highest-ranked men and women per identified era. WRs were not included in the calculations for the pre-IAAF era; XC scores are not applicable for the postwar amateur era; WC and WIC scores are not applicable for the pre-IAAF, interwar, and postwar amateur eras

Rank	Athlete	OG score	WR score	WC score	XC score	WIC score	Total score
**Men**							
*Early era (1896–1912)*							
1	H. Hillman (USA)	350					350
2	W. Tewksbury (USA)	331					331
3	H. Kolehmainen (FIN)	300			30		330
*Interwar era (1920–1936)*							
1	P. Nurmi (FIN)	550	352		60		962
2	V. Ritola (FIN)	400	105		15		520
3	V. Iso-Hollo (FIN)	281	120				412
*Postwar amateur era (1948–1968)*							
1	E. Zátopek (TCH)	450	212				662
2	V. Kuts (URS)	200	224				424
3	P. Snell (NZL)	300	112				412
*Early professional era (1972–1992)*							
1	C. Lewis (USA)	350	117	232			699
2	L. Virén (FIN)	400	205				605
3	S. Coe (GBR)	300	236				536
*Contemporary era (1996–2024)*							
1	U. Bolt (JAM)	600	278	547			1425
2	K. Bekele (ETH)	350	264	372	180	40	1206
3	H. Gebrselassie (ETH)	200	336	372	10	160	1078
**Women**							
*Interwar (1928–1936)*							
1	S. Walasiewicz (POL)	150	260				410
2	L. Radke (GER)	100	134				234
3	B. Didrikson (USA)	100	113				213
*Postwar amateur (1948–1968)*							
1	I. Szewińska (POL)	312	336				648
2	F. Blankers-Koen (NED)	300	226				526
3	S. Strickland (AUS)	293	215				508
*Early professional (1972–1992)*							
1	M. Ottey (JAM/SLO)	255		180		355	790
2	G. Devers (USA)	200		350		200	750
3	T. Kazankina (URS)	300	363	22	15		700
*Contemporary era (1996–2024)*							
1	F. Kipyegon (KEN)	350	204	455			1009
2	T. Dibaba (ETH)	393	124	385	105		1007
3	S.-A. Fraser-Pryce (JAM)	331		477		40	848

OG, Olympic Games; WR, World Record; WC, World Athletics Championships; XC, cross country (includes Olympic Games and World Cross Country Championships); WIC, World Athletics Indoor Championships

The results for each event grouping are presented in Tables 5 (men) and 6 (women). The points do not always match because the event-specific table features points accumulated over those distances only. The number of doubles achieved for the most popular event pairings are presented in Table 7. There were also two doubles achieved in the women’s 1500 m and 3000 m (1983 and 1987 WCs), where the 3000-m WR mean speed was 94.2% and 103.7% of the 1500-m and 5000-m WR speeds, respectively, and in the women’s 1500 m and 10,000 m (Sifan Hassan in the 2019 WC).

**Table 5:** The three highest-ranked men per running discipline. The distances shown in brackets indicate the range of distances (shortest to longest) included in each discipline

Rank	Athlete	OG score	WR score	WC score	XC score	WIC score	Total score
	Sprints (60 m–400 m)						
1	U. Bolt (JAM)	600	278	547			1425
2	M. Johnson (USA)	300	261	420			981
3	C. Lewis (USA)	350	117	232			699
	Hurdles (110 m–400 m)						
1	E. Moses (USA)	231	137	140			508
2	K. Warholm (NOR)	150	113	210			473
3	A. Johnson (USA)	100	0	302			402
	Middle-distance (800 m–1500 m)						
1	H. El Guerrouj (MAR)	250	154	350			754
2	S. Coe (GBR)	300	236	0			536
3	D. Rudisha (KEN)	200	135	140			475
	Steeplechase (2500 m–4000 m)						
1	E. Kemboi (KEN)	200	0	385			585
2	M. Kiptanui (KEN)	50	208	245			503
3	S. El Bakkali (MAR)	200	0	232			432
	Long-distance (5000 m–10,000 m)						
1	K. Bekele (ETH)	350	264	372			986
2	H. Gebrselassie (ETH)	200	336	372			908
3	M. Farah (GBR)	400	0	490			890
	Marathon						
1	A. Bikila (ETH)	200	116	0			316
2	E. Kipchoge (KEN)	200	110	0			310
3	W. Cierpinski (GDR)	200	0	22			222
	Cross country						
1	K. Bekele (ETH)				180		180
2	P. Tergat (KEN)				160		160
3	J. Ngugi (KEN)				150		150
	Indoors (60 m–3000 m)						
= 1	H. Gebreselassie (ETH)					160	160
= 1	B. Lagat (KEN/USA)					160	160
3	A. Johnson (USA)					140	140

OG, Olympic Games; WR, World Record; WC, World Athletics Championships; XC, cross country (includes World Cross Country Championships only); WIC, World Athletics Indoor Championships

**Table 6:** The three highest-ranked women per running discipline. The distances shown in brackets indicate the range of distances (shortest to longest) included in each discipline

Rank	Athlete	OG score	WR score	WC score	XC score	WIC score	Total score
	Sprints (100 m–400 m)						
1	S.-A. Fraser-Pryce (JAM)	331	0	477			808
2	A. Felix (USA)	281	0	359			640
3	F. Griffith-Joyner (USA)	250	353	35			638
	Hurdles (80 m–400 m)						
1	S. McLaughlin (USA)	200	113	105			418
2	D. Muhammad (USA)	150	102	162			414
3	S. Pearson (AUS)	150	0	175			325
	Middle-distance (800 m–1500 m)						
1	F. Kipyegon (KEN)	350	204	455			1009
2	T. Kazankina (URS)	300	363	0			663
3	C. Semenya (RSA)	200	0	232			432
	Steeplechase (3000 m)						
1	G. Galkina-Samitova (RUS)	100	131	22			253
2	B. Chepkoech (KEN)	0	112	105			217
3 =	H. Ghribi (TUN)	100	0	105			205
3 =	W. Yavi (BRN)	100	0	105			205
	Long-distance (3000 m–10,000 m)						
1	T. Dibaba (ETH)	393	124	385			902
2	B. Chebet (KEN)	200	202	197			599
3	M. Defar (ETH)	250	104	219			573
	Marathon						
1	C. Ndereba (KEN)	100	102	175			377
2	P. Jepchirchir (KEN)	100	102	70			270
3	G. Waitz (NOR)	50	106	70			226
	Cross country						
1	G. Waitz (NOR)				170		170
2	L. Jennings (USA)				115		115
3	G. Wami (ETH)				110		110
	Indoors (60 m–3000 m)						
1	M. Mutola (MOZ)					310	310
2	M. Defar (ETH)					210	210
= 3	G. Devers (USA)					200	200
= 3	G. Dibaba (ETH)					200	200

OG, Olympic Games; WR, World Record; WC, World Athletics Championships; XC, cross country (includes World Cross Country Championships only); WIC, World Athletics Indoor Championships

**Table 7:** Similarities and differences between running events paired by “doubles,” where athletes race two events at a single championships. The difference in energy contribution through the oxidative pathway refers to the percentage increase in the aerobic system’s proportion to total energy in the longer of the two events compared with the shorter one it is paired with (men’s data only [25–27])

Event double	Increase in WR duration from shorter to longer event (%)	WR speed of longer event relative to shorter event (%)	Difference in energy contribution through oxidative pathway (%)	Similar events currently available	Number of double victories in the last two eras
100 m/200 m	Men—100.3 Women—103.4	Men—99.8 Women—98.3	100	60 m WIC	Men—OG: 6, WC: 7 Women—OG: 4, WC: 4
200 m/400 m	Men—124.2 Women—123.1	Men—89.2 Women—89.7	48.3	400 m WIC	Men—OG: 1, WC: 1 Women—OG: 2, WC: 0
400 m/800 m	Men—134.5 Women—138.0	Men—85.3 Women—84.0	53.5	400 m/800 m WIC	Men—OG: 1, WC: 0 Women—OG: 0, WC: 1
800 m/1500 m	Men—114.1 Women—102.2	Men—91.9 Women—92.9	27.3	800 m/1500 m WIC	Men—OG: 0, WC: 1 Women—OG: 3, WC: 0
1500 m/5000 m	Men—249.7 Women—267.4	Men—90.9 Women—90.7	12.1	1500 m/3000 m WIC	Men—OG: 1, WC: 1 Women—OG: 0, WC: 1
5000 m/10,000 m	Men—108.0 Women—106.4	Men—96.2 Women—96.9	4.9	3000 m WIC and WXC	Men—OG: 6, WC: 3 Women—OG: 3, WC: 3
10,000 m/marathon	Men—360.5 Women—370.0	Men—91.6 Women—89.8^*^	3.0	WXC	Men—OG: 0, WC: 0 Women—OG: 0, WC: 0

OG, Olympic Games; WC, World Athletics Championships; WXC, World Cross Country Championships; WIC, World Athletics Indoor Championships

*WR achieved in women-only competition

The results of the sensitivity analysis (Table 8) showed that, regardless of which of the chosen scenarios were tested for the normative scoring system, Bolt and Kipyegon were always the respective male and female GOATs. Indeed, the rankings for the men were mostly the same regardless of which points system was used, with all top 10 athletes remaining in the list under all scenarios, but it was noticeable that Gebrselassie moved immediately above Bekele when no XC medals were included. By contrast, Gebrselassie’s position dropped two places when no WIC medals contributed to the overall score. Nurmi was not affected by the removal of XC points, despite winning two Olympic gold medals in the event, but did move up two places when the OG and WR were awarded more points. In women, the same increase in OG and WR points meant that Tatyana Kazankina moved up four places, but the top four places were very fixed in general. Several of the current top 10 women dropped from these positions when the WIC was excluded, as six of this group of women won 33 indoor medals between them.

**Table 8:** The ten highest-ranked men and women used to determine the male GOAT under different scenarios: using the current points system; with OG and WR receiving 140 points; with no bonus for setting a WR at the OG; no bonus for WR duration; no points for XC medals; no points for WIC medals

Rank	Current	OG/WR = 140	No OG + WR bonus	No WR duration	No XC	No WIC
*Men*						
1	Bolt	Bolt	Bolt	Bolt	Bolt	Bolt
2	Bekele	Bekele	Bekele	Bekele	Gebrselassie	Bekele
3	Gebrselassie	Nurmi	Gebrselassie	Gebrselassie	Bekele	Johnson
4	Johnson	Gebrselassie	Johnson	Johnson	Johnson	Nurmi
5	Nurmi	Johnson	Nurmi	Nurmi	Nurmi	Gebrselassie
6	Farah	Farah	Farah	Farah	Farah	Farah
7	El Guerrouj	El Guerrouj	El Guerrouj	El Guerrouj	El Guerrouj	Cheptegei
8	Cheptegei	Cheptegei	Cheptegei	Cheptegei	Cheptegei	El Guerrouj
9	Lewis	Zátopek	Lewis	Lewis	Lewis	Lewis
10	Zátopek	Lewis	Zátopek	Zátopek	Zátopek	Zátopek
*Women*						
1	Kipyegon	Kipyegon	Kipyegon	Kipyegon	Kipyegon	Kipyegon
2	Dibaba	Dibaba	Dibaba	Dibaba	Dibaba	Dibaba
3	Fraser-Pryce	Hassan	Fraser-Pryce	Fraser-Pryce	Fraser-Pryce	Fraser-Pryce
4	Hassan	Fraser-Pryce	Hassan	Hassan	Hassan	Hassan
5	Ottey	Kazankina	Ottey	Ottey	Ottey	Kazankina
6	Defar	Defar	Defar	Defar	Defar	Chebet
7	Devers	Ottey	Devers	Devers	Devers	Kirszenstein
8	Mutola	Kirszenstein	Mutola	Mutola	Mutola	Felix
9	Kazankina	Devers	Kazankina	Kazankina	Kazankina	Griffith-Joyner
10	Chebet	Chebet	Chebet	Chebet	Kirszenstein	Ottey

OG, Olympic Games; WR, World Record; WC, World Athletics Championships; XC, cross country (includes Olympic Games and World Cross Country Championships); WIC, World Athletics Indoor Championships

## Discussion

The aim of this study was to determine the identity of the running GOAT by analyzing historical performance data of running events, which included the allocation of OG and WC medals and setting of WRs. As of March 2026, the male GOAT was Bolt, with a score of 1425, who won medals over 100 m and 200 m and achieved both the highest OG and highest WC scores. Bolt’s overall score for WRs was the fifth highest, although no athlete ranked above him is still a WR holder, and therefore his overall lead could increase over time. Nurmi’s results are worth noting as he placed fifth overall solely on the basis of OG and WR points and did not have the opportunity to compete in WC events; although we did include his OG cross-country medals, the sensitivity analysis showed that he would still have ranked fifth without these, highlighting his exceptionalism. Kipyegon was the GOAT amongst women, with a score of 1009 that would place her fourth on a combined table of men and women (incidentally, a combined top-25 GOAT table would comprise 11 men and 14 women). The dominance of Bolt in the men’s top 25 across the three major accomplishments (OG, WC, and WRs) was not reflected to the same extent amongst women: Shelly-Ann Fraser-Pryce was the highest scorer for the WC, with Kazankina the highest ranked for total WR score. Elaine Thompson-Herah had the highest OG score but, with no WR score, ranked only 25th. Interestingly, Florence Griffith-Joyner’s 100-m WR from 1988 is disputed because of debatable wind readings [13, 28]. If that particular WR had not been ratified, a slower time set by Griffith-Joyner would have been the WR, Thompson-Herah would have set a new women’s 100-m WR at the 2020 Tokyo Olympics [13], and her revised ranking would be 16th. Unlike in the men’s events, many of the longest-lasting women’s WRs were set in the 1980s, which preceded the implementation of systematic in- and out-of-competition drug testing and that could have had a larger effect on women’s performances than on men’s [29]. Indeed, it was noticeable that none of the top 16 women ranked by WR score achieved that feat, or won any medals, after 2000. This meant that ten of the top women did not score any points for WRs, whereas seven men of their top 25 did likewise, and the reduced likelihood of new WRs [30] means future athletes have a diminishing chance of becoming the GOAT. Performance-enhancing drugs have been available to some extent since at least the 1930s [31] and continue to have a large effect on performance [32]. Although there are frequently well-founded suspicions about certain athletes or performances, and athletes retain medals that were won before a doping ban, we included all performances that are currently accepted as legal. We recognize that this means that some medalists and WR holders will have benefitted from undetected doping. Future (legal) improvements in performance are likely to result from better understanding of sport-specific demands, improved competition execution, more specific and precise training load prescriptions, improved quality of training, and more professional and healthier lifestyles [33].

The capacity to accumulate medals is enhanced by the ability to “double up” in more than one event. For example, the determination of Bolt’s position as the male GOAT was facilitated by his three Olympic and three WC 100-m/200-m doubles. Similarly, Fraser-Pryce’s ranking of third on the female GOAT list was aided by winning medals at both 100 m and 200 m. It was similarly notable that, over the longer track distances, amongst the top three men and women, Bekele, Gebrselassie, and Dibaba all won medals over both 5000 m and 10,000 m. These specific sprint and long-distance event pairings accounted for 36 of the 52 doubles (69%) achieved at the OG and WC since 1972 and are closer in terms of increase in WR duration and decrease in WR speed (from the shorter to the longer distance) than any other double (Table 7), showing that they were the most closely matched in terms of physiological demands. In addition, Kipyegon’s ranking as the female GOAT was facilitated by her ability to win medals and set WRs at both 1500 m and 5000 m and is the only woman to have completed this double at any major championships. Although the aerobic contribution to the 200 m was modeled to be twice that of the 100 m [25], these sprint events are heavily dependent on anaerobic contributions and all-out pacing [34]. Likewise, the 5000 m and 10,000 m have similar pacing profiles [34], which could explain the high number of doubles achieved [35], whereas the 10,000 m and marathon have similar aerobic contributions, but achieving the double over the two longest distances is very rare. Zátopek, at the 1952 Helsinki OG, was the only athlete to ever win both the marathon and the 10,000 m (as well as the 5000 m). Attempting doubles at major championships is not just a matter of optimal physiological or tactical characteristics; the scheduling of events, and the number of rounds run, can limit an athlete’s range (and highlight why global championship victories are a greater challenge than meet events), and qualifying in the contemporary era to compete in two events presents a considerable challenge. Indeed, Zátopek was able to achieve his unique treble in 1952 because he was already at the Games when he applied to compete in what was his first marathon only days before it was held [36]. Such an opportunity is impermissible in the contemporary era. Clearly, being on the GOAT list selects those athletes who have the combination of optimal physiological capabilities and strong mental drive required to be a World Champion, WR holder, or Olympic medal winner. Athletes who perform successfully for many years likely have a greater capacity to physically cope with the demands of long-term training and racing activity or must have a high mental “need” to achieve victory that is not assuaged by short periods of success [37]. Further qualitative or quantitative research is required to assess which factors set these GOAT athletes apart from their elite rivals who competed with some success, but not to the extent of those who made the top list, potentially for psycho-social reasons.

One element that always needs consideration when determining the GOAT is the difference in competition availability and standards over that sport’s history. We recognized that contemporary athletes have the good fortune to compete more frequently because of the presence of WC and meet events (e.g., Diamond League), ease of travel, better sport science support, and economic rewards in sport. These are some factors, amongst others, that might allow for longer careers. We also recognized that, as the size of the competitive universe has increased, one has to be relatively better to achieve an Olympic medal or WR now compared with the earliest editions of the modern OG [24]. Several factors therefore affect our overall rankings: the low participation numbers in early OG, difficulties with ratifying WRs in the absence of modern technology, the later introduction of WC, the disruption of two World Wars, and other political interference [3]. Women’s athletics events were particularly restricted in the earliest OG, held back by fabricated media stories of nonfinishers and exhausted competitors in the 1928 Olympic 800-m final, for example [38]. On a greater scale, women’s sport has been limited by social, cultural, and financial restrictions, lack of opportunity, and national gender norms [39]. From the perspective of global athletics success, women who have children during their careers are clearly affected during pregnancy, and postpartum elite athletes require time to return to training and competition [40]. Kipyegon, Fraser-Pryce, and Allyson Felix are three examples from the top 25 ranked women who had children during their competitive careers [40], and clearly this is a consideration when considering their capacity to compete successfully.

Although we did not adjust the normative points system or carry out mathematical or statistical modeling to account for the number of competing athletes, we note that participation has grown substantially since the first modern OG in 1896 (and even the first WC in 1983) and so we split the lifespan of the OG into five separate eras. In general, total points increased with the chronological progression of these eras, as more opportunities to compete and win medals appeared. Our decision to use modern day World Athletics prize money as a system for allocating points was based on its position as an external, widely understood reference for athletic achievement (especially when comparing the color of medals won and the different global competitions held) rather than as a monetary evaluation of the athletes’ performances, given that the timeframe for our study covered more than 125 years and included the amateur eras when runners such as Nurmi competed. Nurmi stands out as an exception in our consideration of the GOAT; he won Olympic medals across the 1920, 1924, and 1928 Games in the 1500 m, 3000-m steeplechase, 5000 m, 10,000 m, and cross country, as well as setting WRs at 1500 m, 5000 m and 10,000 m (the latter of which stood for 15 years). Indeed, if only OG and WR were considered, he would have been, unequivocally, determined to be the male GOAT, and even the removal in the sensitivity analysis of his XC points from the 1920 and 1924 OGs did not affect his overall fifth-place ranking.

Both men’s and women’s top 25 rankings show that several different disciplines were represented. However, those specializing in a single event, such as the steeplechase, marathon, or cross country, were less likely to appear in the overall GOAT top 25. We included the three editions of the Olympic cross-country race, even though there was a ~ 50-year gap between Olympic and WXC events, as it represents a distinct type of running. As a counterbalance to those long-distance athletes competing over the country, we included the WIC for those competing in sprints, hurdles, and middle distances. Only four men and eight women of the whole sample of 2118 athletes scored in both, showing the division between these two off-season championships. Smaller points, as reflected in the most recent prize money on offer, were awarded for these competitions given that they are typically not an athlete’s focus, but we felt they were important to incorporate given they complement the larger global championships within an all-year racing calendar and did boost several of the top 25 athletes’ overall scores, especially in the women’s rankings.

### Limitations

Identifying the GOAT is difficult given the constantly changing sporting, technological, and political landscape. We restricted our analysis to running events, so those athletes who excelled in other athletics events (e.g., Lewis won four Olympic and two World Championship gold medals in the long jump) or in other sports (e.g., “Babe” Didrickson, who was the third-ranked woman in the interwar era) [41] had an even wider span than that covered in our study. One reason for allocating the athletes to different eras was to account for changes in technology, such as the first adoption of synthetic tracks and electronic timing at the 1968 OG [23]. However, we did not adjust points for these changes or the recent emergence of advanced footwear technology (“super shoes”), which have increasingly been adopted since 2016 [42], and are now one element of developing technology perhaps required to break WRs [43]. Allocating athletes to eras also allows for a better appreciation of the achievements of athletes in earlier eras when there were fewer competitive opportunities (of the type analyzed in our study), as the overall normative scoring system does not account for either the lack of opportunities in earlier eras or the increased number of nations now competing in global competition (e.g., after the breakup of states like the Soviet Union and Yugoslavia). We restricted our analysis to Olympic and World Athletics competitions, as these were global events that member federations could enter qualified athletes for (rather than those competitions where participation was by invitation only). We consequently omitted performances in meet events such as the Diamond League (where distance WRs are more frequent because of pacemakers and pacing lights) [44], Area championships (e.g., European Championships), age-group competition, road races, and big-city marathons. The latter is particularly important as many runners favor participation in big-city marathons, where WRs are largely set, rather than in championships. This did mean excluding the many large-city and World Marathon Major victories of athletes such as Waitz (11 wins), Eliud Kipchoge (11 wins), and Paula Radcliffe (8 wins) and, by the same token, we also did not include victories for track athletes in present or historical major meet series, such as the IAAF Golden League. Athlete appearances in these events are typically by invitation and do not require the championship approach required to progress through qualifying rounds over a short period. Even though we did factor in WR duration, we did not consider competitive dominance in other ways, such as winning streaks. Although we have identified the GOAT in both sexes as well as categories of GOAT across eras and events using a normative points system based on current World Athletics prize money, there are always external factors that cannot be easily quantified or modeled, but which could be an interesting concept for future research. Nonetheless, the process we adopted forms a robust, quantitative starting basis on which further debate and analysis can be developed.

## Conclusions

In summary, using a clearly defined points system for scoring athletes who were serial winners, we have described those whom we have identified as the GOAT runners both overall and separately by sex, distance, and type of running event. Using this approach, Bolt and Kipyegon were determined to be the men’s and women’s GOAT, respectively. Although no manner of performing such a ranking task can be perfect or completely objective, we believe that both the GOATs chosen and the way they were chosen will stimulate interest in those working in the field to develop more robust methods or validate our findings. The model created can be used in future years when athletes achieve successes that break current WRs or overtake the breadth and standard of success of the current GOAT runners identified. Each of these athletes are remarkable for their achievements, and further work is needed to better understand how they perform at a competitive standard that stands out even amongst their elite peers at the pinnacle of their sport.

## Supplementary Information

Below is the link to the electronic supplementary material.

Supplementary file1 (XLSX 563 KB)

## Declarations

### Availability of Data and Material

All data are provided within the main manuscript and from a publicly available dataset (https://figshare.leedsbeckett.ac.uk/articles/dataset/Running_Greatest_Of_All_Time_-_dataset/29364917).
